# Informal employment, precariousness, and decent work: from research to preventive action

**DOI:** 10.5271/sjweh.4024

**Published:** 2022-03-31

**Authors:** Fernando G. Benavides, Michael Silva-Peñaherrera, Alejandra Vives

**Affiliations:** 1 Center for Research in Occupational Health, Universitat Pompeu Fabra, Barcelona, Spain; 2 Ibero-American Observatory on Safety and Health at Work, Ibero-American Social Security Organization; 3 CIBER Epidemiología y Salud Pública (CIBERESP) and IMIM (Hospital del Mar Medical Research Institute), Barcelona, Spain; 4 Departamento de Salud Pública, Pontificia Universidad Católica de Chile. CEDEUS. Santiago, Chile

Decent work, defined by the International Labor Organization (ILO) in 1999 in relation to promoting productive and freely chosen employment, fair income, security in the workplace, guaranteeing rights at work, granting social protection, freedom of organization and creating social dialogue, is a basic condition for the health and wellbeing of workers and their families (1). Underpinned by the Declaration of Philadelphia, which states that all human beings have the right to develop in freedom and dignity, economic security and equal opportunity (2), decent work is key goal number 8 of the United Nations’ 2030 Agenda for Sustainable Development (SDG).

However, informal employment – that is work without a contract, legal protection or social security – is the most common employment arrangement in the world. According to the ILO, its share of total employment (SDG indicator 8.3.1) was 61% (2 billion workers) by 2016. While globally the proportion of informal workers is larger among men (63% versus 58% for women), there are more countries (55%) where the share of women in informal employment exceeds that of men (3). In fact, informal employment constitutes a persistent, structural pillar of labor markets, especially in low- and middle-income countries. And while in the USA and the EU, informal employment is around 18% and 15% of the occupied labor force, respectively, figures are 53% for Latin America, and 88% and 77% in Africa and Asia (excluding China), respectively.

Despite this overwhelming reality, we have little evidence of the health impact of informal employment. In a seminal study carried out in Brazil, Santana & Loomis (4) showed that workers without job contracts (informal employment) had a 20% greater risk of occupational injury than workers with a contract (formal employment). In a recent 2011 study based on the first working and employment conditions survey in Central America, Lopez-Ruiz et al (5) showed that the lack of social security coverage (informal employment) was associated with a higher prevalence of poor self-perceived health and poor mental health in both genders. Utzet et al (6) confirmed these results in a study based on a sample of 176 786 workers from national working conditions and health surveys carried out in 13 Latin American and Caribbean countries between 2012 and 2018, which found that informal workers (defined by coverage or contribution to a health/pension plan/insurance) reported significantly worse self-perceived health than formal workers, both among women [1.28 (95% CI 1.14–1.43)] and men [1.30 (1.12-1.50)]. In another study, based on ecological data (2000–2016) for 17 Latin American countries, Silva-Peñaherrera et al (7) found a clear and significant positive association between the national rates of informal employment and adult mortality rates.

The main hypothesized mechanisms that could explain the relationship between informal employment and poor health include poor employment conditions (low income, employment insecurity, lack of social security and workplace rights, etc.) and poor working conditions (unsafe workplace, poor air quality, heavy load handling, high demand and low control, etc.), as well as poor living conditions and limited access to health services. However, much research is still needed to better understand the health consequences of informal employment and its mechanisms, acknowledging that informal work is a heterogeneous phenomenon that makes both research and policy-making especially challenging.

In high-income countries, on the other hand, where informal employment is generally <20% (8), its effect on worker’s health has been studied scarcely (9) and with contradictory results. For example, a study using the European Working Conditions Survey of 2010 did not find differences in self-perceived health between formal and informal employees (10), while another study conducted with data from Spain comparing four different employment profiles only found differences in self perceived health status among women in informal employment (11). An explanation for these results could be that healthcare coverage in Spain is universal, and some social protection services, not necessary by direct contribution to the social security system, are not restricted to formal employees. This might attenuate possible health inequalities resulting from working in informal arrangements. In Chile, using the 2010 employment conditions survey, Ruiz et al (12) found differences in health that were only significant among men. The authors hypothesized that formalization may not be as beneficial for women as men given the higher levels of precariousness of female formal employment and the double burden of unpaid work.

Conversely, research on the health impact of precarious employment on formally employed workers have increased in the last two decades (13). In fact, precarious employment, which may affect the full range of standard and non-standard employment arrangements, is considered an emerging determinant of health (14). One reason that could explain this interest, as compared to the volume of research on informal employment and health, is that employment precariousness is a growing problem in high income countries (15, 16). This progress in the available evidence on the association between precarious employment and poor health is also not unrelated to the fact of having a developed a conceptual proposal of the key dimensions of employment precariousness for health, and a measurement instrument, the Employment Precariousness Scale – EPRES (17), which has been adapted to different countries from Chile (18), Spain (19), Sweden (20) and Greece (21) to the European Union (22) more recently. Ervasti & Virtanen (23) has underlined these efforts and their limits.

Briefly, we could summarize that informal employment is characterized by the lack of a contract and social security coverage and that precarious employment is characterized by a weak employment relation with different extents of employment insecurity, low wages, limited workplace rights and social protection among formally employed workers. A shared feature of workers in poor employment conditions is that they generally perform less qualified jobs, face more risks at work and lack the power to bargain over their working and employment conditions to improve them (24). Informal and precarious workers are generally invisible for statistics, such as occupational injury and unemployment rates and, as a consequence, do not exist for policy-making or social security purposes. Globally the gap is enormous: only 35% of the world’s employed population has coverage for occupational injuries and <19% of the unemployed receive unemployment benefits (25).

To manage this social interaction, we propose a unified conceptual framework of working and employment conditions. For public policy decision-makers and professional practitioners, the former are the classical object of the occupational health and safety issue, and the latter are the object of the human resources issue. Integrating both dimensions in an axis of coordinates, as is shown in figure 1, we could broaden our understanding of decent work as the best interaction between working and employment conditions. In this scenario, informal employment generally represents the worse scenario as unprotected jobs lack employment and income security and are performed in unhealthy and unsafe working conditions. In the middle position would be precariously employed salaried workers, who – although covered by legal regulatory frameworks – are frequently ill-protected by labor inspection, granted limited or insufficient social security coverage, and most probably incapable of demanding healthy working conditions. Finally, the best and desirable scenario would be decent work combining healthy and nurturing employment and working conditions.

**Figure 1:**
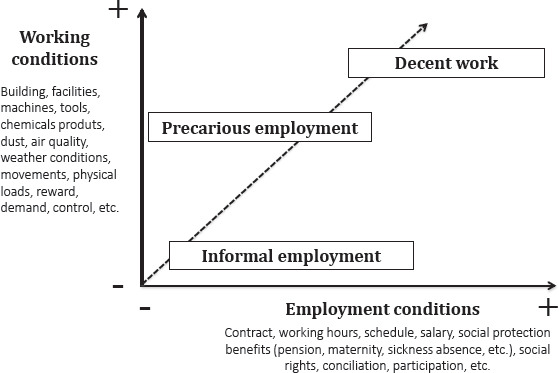
Conceptual framework of the interaction between employment and working conditions for health: moving from informal and precarious employment to decent work.

This framework may contribute to understanding that employment and working conditions are strongly interrelated and that both require attention both from research and policy making. The ILO emphasizes that promoting decent work for all requires realizing fundamental rights at work, creating more and better employment and income opportunities, extending social protection, and promoting social dialogue. We add that this is not only a necessary step towards decent employment but an indispensable one to grant all workers adequate working conditions and thus achieve the goal of decent work in an integral manner.

We are in the middle of a great transformation probably comparable to the Industrial Revolution in the 18^th^ and 19^th^ centuries. One which has been accelerated by the COVID-19 pandemic (26). An epoque change characterized by a new economic and labor space, as well as a social one – cyberspace – which deepens the digitization of the economy and the exponential increase in the flexibility of the labor market (27). Think of gig-working in delivery or driving, at a high risk for workers own health, or other forms of platform teleworkers, caregivers, etc., always available for the work that is needed, when it is needed. It is the other side of the relocation of work, both physically and organizationally, and with the only link being the internet connection (28). New forms of work on the bridge between self-employment and informal or highly precarious dependent work that require still greater effort if the health of the workforce is to be protected.

## References

[1] The ILO's Decent Work Agenda.

[2] International Labour Organization R204 - Transition from the Informal to the Formal Economy Recommendation, 2015 (No. 204).

[3] International Labour Organization (2018). Women and men in the informal economy:a statistical picture (third edition).

[4] Santana V, Loomis D (2004). Informal jobs and non-fatal occupational injuries. Ann Occup Hyg.

[5] Lopez-Ruiz M, Benavides FG, Vives A, Artazcoz L (2017). Informal employment, unpaid care work, and health status in Spanish-speaking Central American countries:a gender-based approach. Int J Public Health.

[6] Utzet M, Botías F, Silva-Peñaherrera M, Tobías A, Benavides FG (2021). Informal employment and poor self-perceived health in Latin America and the Caribbean:a gender-based comparison between countries and welfare states in a pooled analysis of 176,786 workers. Global Health.

[7] Silva-Peñaherrera M, López-Ruiz M, Merino-Salazar P, Gomez-Garcia A, Benavides FG (2021). Association between informal employment and mortality rate by welfare regime in Latin America and the Caribbean:an ecological study. BMJ Open.

[8] OECD/ILO (2019). Tackling Vulnerability in the Informal Economy, Development Centre Studies.

[9] Julia M, Tarafa G, O'Campo P, Muntaner C, Jodar P, Benach J (2016). Informal employment in high-income countries for a health inequalities research:a scoping review. Work.

[10] Julia M, Belvis F, Vives A, Tarafa G, Benach J (2019). Informal employees in the European Union:working conditions, employment precariousness and health. J Public Health.

[11] Montero-Moraga JM, Benavides FG, Lopez-Ruiz M (2020). Association between informal employment and health status and the role of the working conditions in Spain. Int J Health Serv.

[12] Ruiz M, Vives A, Martínez-Solanas E, Julià M, Benach J (2017). How does informal employment impact population health?Lessons from the Chilean employment conditions survey. Safety Science.

[13] Rönnblad T, Grönholm E, Jonsson J, Koranyi I, Orellana C, Kreshpaj B (2019). Precarious employment and mental health:a systematic review and meta-analysis of longitudinal studies. Scand J Work, Environ Health.

[14] Benach J, Vives A, Amable M, Vanroelen C, Tarafa G, Muntaner C (2014). Precarious employment:understanding an emerging social determinant of health. Annu Rev Public Health.

[15] Quinlan M, Mayhew C, Bohle P (2001). The global expansion of precarious employment, work disorganization, and consequences for occupational health:placing the debate in a comparative historical context. Int J Health Services.

[16] Benach J, Vives A, Tarafa G, Delclos C, Muntaner C (2016). What should we know about precarious employment and health in 2025?Framing the agenda for the next decade of research. Int J Epidemiol.

[17] Vives A, Amable M, Ferrer M, Moncada S, Llorens C, Muntaner C, Benavides FG, Benach J (2010). The Employment Precariousness Scale (EPRES):psychometric properties of a new tool for epidemiological studies among waged and salaried workers. Occup Environ Med.

[18] Vives A, González F, Solar O, Bernales P, González MJ, Benach J (2017). Precarious employment in Chile:psychometric properties of the Chilean version of Employment Precariousness Scale in private sector workers. Cad Sau´de Pu´blica.

[19] Vives A, Gonzalez F, Moncada S, Llorens C, Benach J (2015). Measuring precarious employment in times of crisis:the revised Employment Precariousness Scale (EPRES) in Spain. Gac Sanit.

[20] Jonsson J, Vives A, Benach J, Kjellberg K, Selander J, Johansson G (2019). Measuring precarious employment in Sweden:translation, adaptation and psychometric properties of the Employment Precariousness Scale (EPRES). BMJ Open.

[21] Tsopoki Vassiliki M, Sourtzi P, Galanis P, Vives A, Benach J, Tziaferi S, Velonakis E (2019). Cross-cultural adaptation and validation of the Employment Precariousness Scale (EPRES) in employees in Greece. Nurs Care Research.

[22] Padrosa E, Vanroelen C, Muntaner C, Benach J, Julià M (2022). Precarious employment and mental health across European welfare states:a gender perspective. Int Arch Occup Environ Health.

[23] Ervasti J, Virtanen M (2019). Research strategies for precarious employment. Scand J Work Environ Health.

[24] Benach J, Vives A, Amable M, Vanroelen C, Tarafa G, Muntaner C (2014). Precarious employment:understanding an emerging social determinant of health. Ann Rev Public Health.

[25] ILO Data Daschboard on Universal Social Protection.

[26] ILO Global Summit on COVID-19 and the World of Work Concept note.

[27] Rani U, Kumar Dhir R, Furrer M, Gőbel N, Moraiti A, &Cooney S (2021). World Employment and Social Outlook:The Role of Digital Labour Platforms in Transforming the World of Work.

[28] Jetha A, Shamaee A, Bonaccio (2021). Fragmentation in the future of work:A horizon scan examining the impact of the changing nature of work on workers experiencing vulnerability. Am J Ind Med.

